# Refractory multiple brain abscesses caused by *Prevotella loescheii* and *Porphyromonas gingivalis*: successful endoscopic lavage and drainage: a case report and review of the literature

**DOI:** 10.3389/fmed.2026.1736006

**Published:** 2026-02-03

**Authors:** Lirui Dai, Wenyi Zhan, Xinyuejia Huang, Liang Lyu, Shu Jiang, Peizhi Zhou

**Affiliations:** Department of Neurosurgery, West China Hospital of Sichuan University, Sichuan University, Chengdu, Sichuan, China

**Keywords:** brain abscess, endoscopic lavage, metagenomic next-generation sequencing (mNGS), odontogenic origin, *Porphyromonas gingivalis*, *Prevotella loescheii*

## Abstract

**Background:**

Brain abscesses represent life-threatening conditions, with management complexities significantly heightened in cases involving multiple lesions that are refractory to standard empirical therapies. *Prevotella loescheii* and *Porphyromonas gingivalis*, anaerobic bacteria typically residing within the oral flora, are infrequent yet formidable pathogens responsible for intracranial abscess formation. The fastidious nature of these microorganisms often results in delayed diagnosis and initiation of targeted treatment.

**Case presentation:**

A 25-years-old male presented with a 1-month history of cough, sputum production, and persistent high-grade fever reaching 41 °C. Initially diagnosed with a brain abscess at a local hospital, he received empirical treatment with ceftriaxone, acyclovir, and mannitol, which failed to yield clinical improvement. His condition subsequently deteriorated, characterized by disturbances in consciousness and dysarthria. The antimicrobial regimen was escalated to include vancomycin and meropenem. Despite these efforts, the patient’s neurological status continued to decline, with imaging studies revealing the development of multiple new intracranial abscesses and diffuse intracranial hypertension. Surgical intervention was undertaken, involving abscess excision and decompressive craniectomy. Postoperative imaging 1 week later showed further abscess expansion and the onset of right-sided hemiplegia. Upon admission to our institution, metagenomic next-generation sequencing (mNGS) of the cerebrospinal fluid identified the presence of *Prevotella loescheii* and *Porphyromonas gingivalis*. The antimicrobial regimen consisting of vancomycin and meropenem was maintained, and the patient underwent endoscopic intracranial abscess lavage with burr hole external drainage. This integrated approach led to significant radiographic resolution of the abscesses and a gradual improvement in the patient’s level of consciousness. The refractory infection was traced back to an oropharyngeal source.

**Conclusion:**

This case highlights the critical diagnostic value of mNGS in detecting fastidious oral anaerobic pathogens in culture-negative refractory brain abscesses. It illustrates that a combination of targeted antibiotic therapy and minimally invasive surgical intervention–specifically, endoscopic lavage and drainage–can be highly effective in managing complex, multi-loculated abscesses caused by *Prevotella loescheii* and *Porphyromonas gingivalis*. Maintaining a high index of suspicion for an odontogenic or oropharyngeal origin is crucial in the diagnostic evaluation of such infections.

## Introduction

Brain abscesses constitute a significant neurosurgical emergency, characterized by localized intracranial purulent collections. Despite notable advancements in diagnostic techniques and antimicrobial treatments, these abscesses remain associated with considerable risks of mortality and long-term neurological complications (1, 2). Effective management of brain abscesses generally necessitates a comprehensive approach, integrating surgical procedures for pathogen identification and abscess drainage with extended antibiotic therapy (3, 4).

The etiology of brain abscesses is frequently associated with contiguous spread from otogenic or odontogenic sources, hematogenous spread from distant locations, or penetrating cranial injuries (5–8). Anaerobic bacteria, especially those originating from the oral cavity, are commonly implicated and present significant diagnostic challenges. *Prevotella loescheii* and *Porphyromonas gingivalis*, which are fastidious, obligate anaerobic Gram-negative bacilli, are prevalent components of the oral microbiota and are well-known contributors to periodontal disease (9, 10). Their isolation and identification through conventional culture methods are notoriously difficult, often leading to delayed diagnosis and inappropriate empirical treatment.

Metagenomic next-generation sequencing (mNGS) has emerged as a robust and unbiased diagnostic modality for the direct identification of pathogens from clinical specimens, demonstrating particular utility in the diagnosis of rare, polymicrobial, or culture-negative central nervous system infections (11, 12). Concurrently, the surgical management of complex, multi-loculated abscesses is undergoing significant advancements. Traditional methods such as excision or simple aspiration remain standard treatments; however, endoscopic lavage and drainage present a minimally invasive yet comprehensive alternative, facilitating the evacuation of pus, disruption of loculations, and debridement of the cavity under direct visualization (13, 14).

In this report, we discuss a challenging case involving a young male patient with refractory, multiple brain abscesses attributed to *P. loescheii* and *P. gingivalis*. This case underscores the critical diagnostic role of mNGS in identifying fastidious pathogens and illustrates the therapeutic efficacy of a combined approach utilizing targeted antibiotic therapy alongside advanced endoscopic surgical techniques.

## Case illustration

### Initial presentation and deterioration at local hospital

The patient, a previously healthy 25-years-old male, initially presented to a local hospital with a 1-month history of productive cough, sputum production, and persistent high-grade fever, with temperatures reaching up to 41 °C. The initial neurological examination was reportedly unremarkable. Cranial magnetic resonance imaging (MRI) identified a solitary lesion in the left frontal lobe, indicative of an early-stage brain abscess. The patient was commenced on empirical intravenous therapy, which included ceftriaxone for broad bacterial coverage, acyclovir for empirical coverage of herpes simplex encephalitis, and mannitol for osmotherapy to manage cerebral edema. Despite this intervention, the patient’s systemic and neurological symptoms did not improve over the subsequent week.

The patient’s condition progressively worsened, characterized by a marked disturbance in consciousness, including drowsiness and confusion, as well as dysarthria. In response to this clinical deterioration, the antimicrobial regimen was intensified to include vancomycin, targeting methicillin-resistant *Staphylococcus aureus*, and meropenem, a broad-spectrum carbapenem. Despite this aggressive empirical treatment, follow-up MRI revealed a concerning progression to multiple new intracranial abscesses in both cerebral hemispheres, accompanied by signs of diffuse intracranial hypertension.

### Initial surgical intervention and subsequent transfer

**FIGURE 1:**
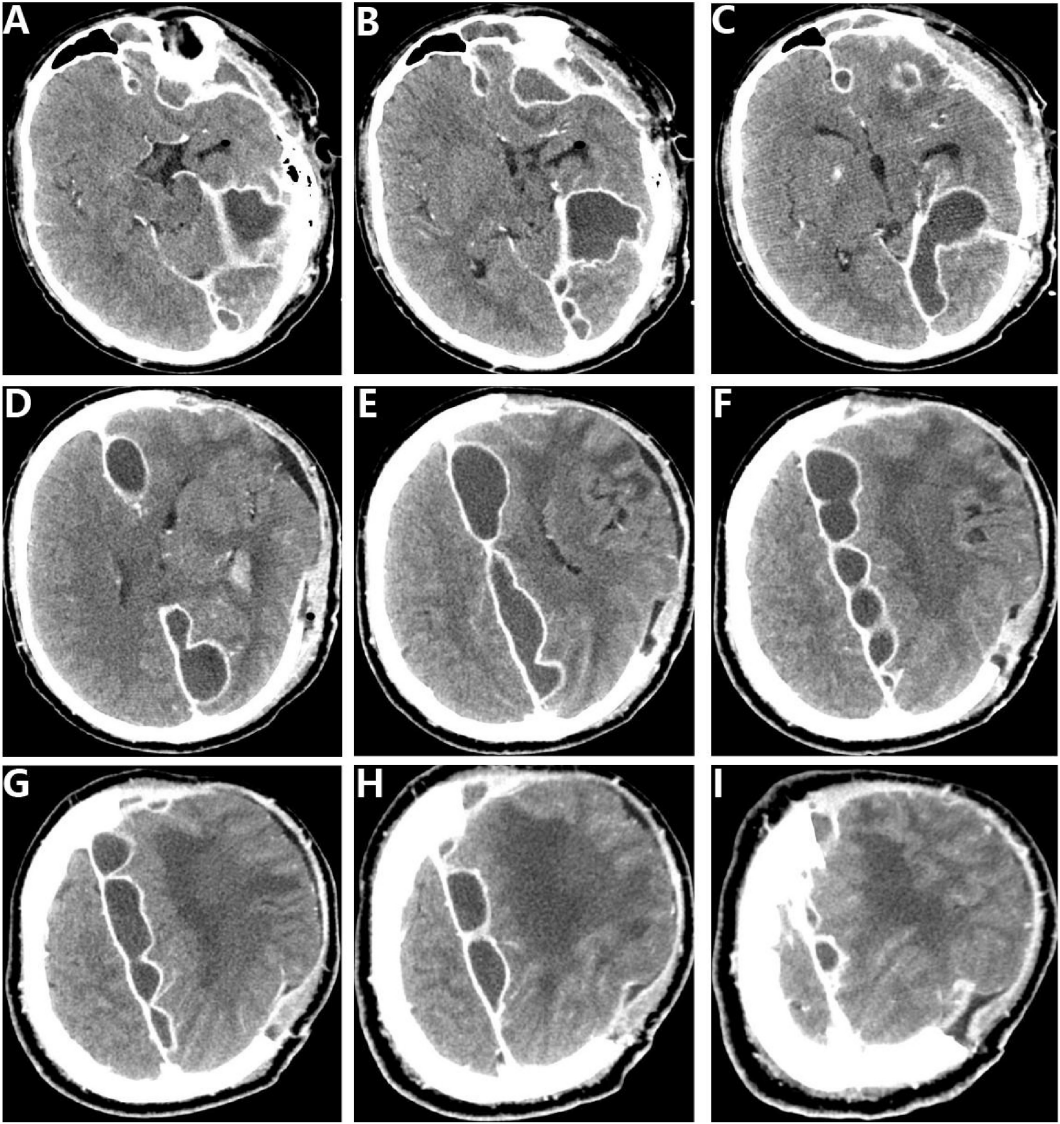
Head enhanced CT prior to neuroendoscopic treatment of the patient (preoperative CT). **(A–I)** Preoperative head CT images of the patient showing the condition of brain abscesses.

In light of the persistent clinical and radiological deterioration, the patient underwent an urgent surgical intervention. This procedure included a craniotomy for the excision of the largest abscesses and a decompressive craniectomy to alleviate life-threatening intracranial pressure. Intraoperative samples were collected for standard bacterial and fungal cultures. Postoperatively, the patient’s neurological condition remained critical. A follow-up computed tomography (CT) conducted 1 week after surgery indicated further expansion of the residual abscesses and the onset of new right-sided hemiplegia (Figure 1). Confronted with this therapeutic impasse, the patient was transferred to our hospital for advanced management.

### Diagnostic re-evaluation and mNGS findings at our institution

Upon admission, the patient presented with fever and was in a comatose state, with a Glasgow Coma Scale score of 6. A comprehensive physical examination revealed poor dental hygiene and signs of chronic periodontitis. We promptly re-evaluated the diagnostic workup. Cerebrospinal fluid (CSF) obtained via lumbar puncture demonstrated a markedly elevated white blood cell count, predominantly neutrophils, and an increased protein level. Given the failure of conventional cultures and the critical nature of the infection, the CSF sample was subjected to mNGS. In this case, mNGS refers to directly obtaining brain abscess specimens from the patient’s brain, thereby extracting all microbial nucleic acids (DNA and/or RNA). Using a high-throughput sequencing platform, millions to billions of nucleic acid fragments are simultaneously analyzed. Then, through bioinformatics algorithms and comparison with known microbial genome databases, possible bacteria, fungi, viruses, parasites, drug resistance genes, and virulence factors in the sample can be detected. The analysis definitively identified nucleic acid sequences corresponding to *Prevotella loescheii* and *Porphyromonas gingivalis*, with a high sequence count for both organisms. No other pathogenic bacteria, viruses, or fungi were detected.

### Targeted surgical management and clinical outcome

Based on the conclusive metagenomic next-generation sequencing (mNGS) results, the existing antibiotic regimen of vancomycin and meropenem was maintained. Meropenem, in particular, offers excellent anaerobic coverage and penetrates effectively into the central nervous system, thereby justifying its continued use against the identified pathogens. Given the failure of previous open surgical interventions to control the disease, an alternative, minimally invasive surgical strategy was adopted.

**FIGURE 2:**
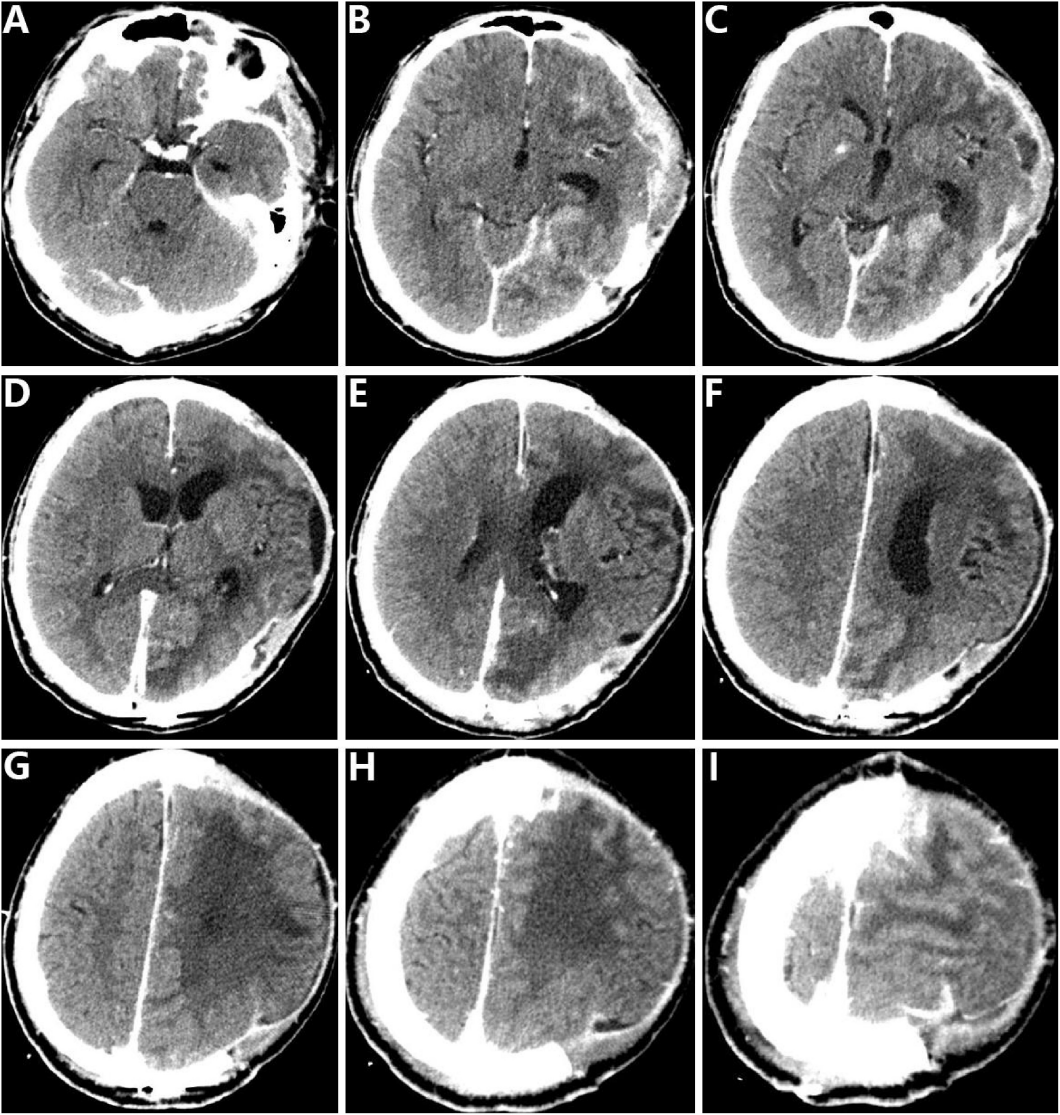
Postoperative head enhanced CT of the patient (postoperative CT). **(A–I)** Postoperative head CT images of the patient showing the condition of brain abscesses.

We conducted endoscopic intracranial abscess lavage in conjunction with burr hole external drainage. This technique employed a rigid neuro-endoscope, which facilitated direct visualization of the abscess cavities, extensive irrigation with saline to disrupt septations and loculations, and complete evacuation of purulent material (Figure 2). In the left frontal, temporal, parietal, and occipital regions of the patient, there are pre-existing surgical incisions. The bone in this area is absent, and intracranial pressure is elevated. The procedure involves incising the skin and subcutaneous tissue along the previous surgical incision, followed by drilling a small hole adjacent to the midline of the skull. A milling tool is then employed to create a small bone window. A fistula, approximately 1 cm in diameter, is made at the superficial part of the abscess to expose the abscess wall. The pus within is noted to be thick and viscous. Approximately 4500 ml of warm normal saline is used for thorough irrigation. A ventriculoscope is inserted to facilitate slow irrigation, gradually advancing into the lateral parieto-occipital abscess cavity. The irrigation is then directed posteriorly along the midline toward the anterior side, with the aim of separating adhesions between the abscess walls until the irrigation fluid becomes clear. Postoperatively, the patient exhibited remarkable and rapid improvement. A follow-up CT scan conducted 1 week later revealed significant resolution of the multiple abscesses and a notable reduction in mass effect and surrounding edema. The patient’s level of consciousness gradually improved, and he began to follow simple commands. The right-sided hemiplegia showed modest improvement with intensive, early physiotherapy. A subsequent formal dental consultation confirmed severe periodontitis, establishing the likely oropharyngeal source of the bacteremia that seeded the brain abscesses. In addition, to prevent epilepsy, the patient was administered levetiracetam 1000 mg twice daily before the surgery. After the patient regained consciousness after the surgery, the medication was changed to levetiracetam tablets 0.75 g twice daily by mouth, and valproic acid sodium 0.4 g was taken four times a day. During the entire treatment process, we used 1000 mg of vancomycin every 12 h and 2 g of meropenem every 8 h to combat infections.

## Discussion

This case provides several significant insights into the diagnosis and management of complex intracranial infections, particularly those caused by fastidious anaerobic bacteria, highlighting the crucial role of modern technologies in guiding treatment toward a successful outcome.

Although *P. loescheii* and *P. gingivalis* are common oral commensals, they are rare etiological agents of severe intracranial infections. These bacteria typically enter the bloodstream through disruptions in the oral mucosa, which can occur due to periodontal disease, dental procedures, or even minor trauma from chewing (15–17). In our case, the initial empirical therapy with ceftriaxone, which has variable anaerobic coverage, and even the escalated regimen of vancomycin and meropenem, failed to halt disease progression. This underscores the difficulty of managing such infections without identifying a definitive pathogen and the potential for initial treatment failure despite broad-spectrum coverage.

The turnaround time for traditional culture methods can range from several days to weeks, with often low yield, particularly in cases where the patient has previously received antibiotics, as was observed in this instance. mNGS has transformed the management of our patient by enabling rapid, comprehensive, and culture-independent identification of the causative pathogens within a few days (18–20). This technology facilitates the unbiased detection of virtually all nucleic acids present in a sample, rendering it exceptionally valuable for diagnosing atypical, polymicrobial, or culture-negative infections (21–23). The precise identification of *P. loescheii* and *P. gingivalis* confirmed the anaerobic and likely odontogenic nature of the infection. This not only validated the continued use of carbapenem therapy but also negated the necessity for additional antifungal or antiviral treatments, thereby allowing for a more targeted and potentially safer therapeutic approach.

The standard care for brain abscesses typically involves surgical drainage or excision in conjunction with antibiotic therapy (24–26). However, the optimal surgical strategy for managing multiple or deep-seated abscesses remains a subject of ongoing debate. The patient’s initial open excision and decompressive craniectomy were insufficient, possibly because the procedure failed to address all loculations and deeply seated pockets of infection adequately, or because it caused significant inflammatory disruption, potentially exacerbating the condition.

Neuro-endoscopy presents a minimally invasive yet highly effective alternative, as underscored by an expanding body of literature. Endoscopic aspiration and lavage facilitate direct visualization, allowing for more comprehensive evacuation of pus from multiple sites, meticulous disintegration of septations, and efficient irrigation of the cavity (27, 28). This technique is associated with reduced cerebral trauma compared to open craniotomy, more thorough debridement than blind aspiration, and potentially lower recurrence rates (29). In our patient, the strategic transition to endoscopic lavage and drainage marked a definitive turning point following the failure of other interventions, resulting in rapid clinical and radiological improvement. This outcome is consistent with reported cases where endoscopic lavage successfully managed severe intracranial infections that were unresponsive to external drainage alone (30).

The initially empirical antibiotic regimen of meropenem and vancomycin was ultimately deemed appropriate. Although metronidazole is frequently regarded as the gold standard for anaerobic infections, carbapenems such as meropenem offer excellent anaerobic coverage and effectively penetrate the central nervous system, rendering them a suitable therapeutic option. The continuation of this regimen, once informed by mNGS, was thus justified. Importantly, the identification of an oral source emphasizes the critical need for comprehensive dental evaluation and subsequent definitive dental care in such patients to prevent recurrence, a step that is occasionally overlooked during the acute management phase.

## Conclusion

This case report presents a severe and life-threatening instance of multiple brain abscesses caused by *Prevotella loescheii* and *Porphyromonas gingivalis* of oropharyngeal origin. It underscores several key considerations for clinicians: (1) Maintain a high index of suspicion: odontogenic pathogens should be included in the differential diagnosis of cryptogenic brain abscesses, particularly in young adults without other risk factors. A thorough oral examination is an essential, non-invasive component of the diagnostic workup. (2) Adopt advanced diagnostic techniques: mNGS is a transformative tool capable of rapidly identifying the etiology of culture-negative, refractory CNS infections. This enables precise and timely antimicrobial therapy, effectively overcoming the limitations associated with conventional microbiological methods. (3) Customize the surgical strategy: in cases of complex, multi-loculated abscesses that are unresponsive to initial drainage, endoscopic lavage and drainage offer a highly effective and minimally invasive surgical option. This approach can provide superior decompression and debridement compared to traditional methods, potentially leading to faster recovery and improved outcomes. Overall, a multidisciplinary approach that integrates neurosurgery, infectious diseases, and dental medicine, guided by rapid molecular diagnostics and advanced surgical techniques, is essential for the successful management of such challenging cases.

## Data Availability

The original contributions presented in this study are included in this article/supplementary material, further inquiries can be directed to the corresponding author.
